# The Remodulation of Actin Bundles during the Stimulation of Mitochondria in Adult Human Fibroblasts in Response to Light

**DOI:** 10.3390/pharmaceutics16010020

**Published:** 2023-12-22

**Authors:** Soňa Olejárová, Denis Horváth, Veronika Huntošová

**Affiliations:** 1 Department of Biophysics, Institute of Physics, Faculty of Science, P.J. Šafárik University in Košice, Jesenná 5, 041 54 Kosice, Slovakia; sona.olejarova@student.upjs.sk; 2 Center for Interdisciplinary Biosciences, Technology and Innovation Park, P.J. Šafárik University in Košice, Jesenná 5, 041 54 Košice, Slovakia; denis.horvath@upjs.sk

**Keywords:** actin, cytoskeleton, photobiomodulation, mitochondria, photodynamic therapy, hypericin, fibroblast, STED microscopy

## Abstract

β-actin belongs to cytoskeletal structures that change dynamically in cells according to various stimuli. Human skin can be considered as an organ that is very frequently exposed to various stress factors, of which light plays an important role. The present study focuses on adult human fibroblasts exposed to two types of light stress. Orange light with a wavelength of 590 nm was used here to stimulate the photosensitizer localized in the cells as a residual dose of photodynamic therapy (PDT). On the other hand, near-infrared light with a wavelength of 808 nm was considered for photobiomodulation (PBM), which is often used in healing processes. Confocal fluorescence microscopy was used to observe changes in intercellular communication, mitochondrial structures, and cytoskeletal dynamics defined by the remodulation of β-actin of fibroblasts. The number of β-actin bundles forming spherical structures was detected after light exposure. These structures as β-actin oligomers were confirmed with super-resolution microscopy. While PDT led to the disintegration of actin oligomers, PBM increased their number. The interaction of β-actin with mitochondria was observed. The combination of PDT and PBM treatments is important to minimize the side effects of cancer treatment with PDT on healthy cells, as shown by the cell metabolism assay in this work. In this work, β-actin is presented as an important parameter that changes and is involved in the response of cells to PDT and PBM.

## 1. Introduction

The cytoskeleton is an important support for cell structure. The versatile functionality of the cytoskeleton as a fibrous complex was recently summarized in a complex review [1]. Its function is involved in processes such as cell division, motility, shape formation, and also endocytosis and intracellular transport. It is composed of three types of proteins: actin, tubulin, and proteins that form intermediate filaments. It has been recognized that β-actin occurs in two forms that are balanced in the actin cytoskeleton [2]. The simple actin filament consists of globular actin (G-actin). G-actin can polymerize into asymmetric helical structures as filamentous actin (F-actin) [3]. Actin is ATPase and, after ATP binding, G-actin can form dimers and trimers depending on its condition [4]. In addition, stable actin oligomers can be found under physiological conditions. Filaments are formed in a right-handed helical structure. Several proteins are involved in the polymerization process that leads to dendritic actin networks and actin bundles and play a role in contractility and disassembly [1].

Actin filament contractility is controlled by myosin family proteins. They are important for movement and interaction with the environment [5]. Energy supply to myosin occurs through ATP hydrolysis, and myosin activation depends on phosphorylation, which can be controlled by kinases and protein kinases, including protein kinase C [6].

In general, cytoplasmic actin regulates cell morphology, movement, and organelle dynamics [7]. The regulation of mitochondrial function can be governed via proteins (e.g., myosin) associated with actin [8,9]. Xie et al. reported in their work that β-actin is required for mitochondrial quality control [10]. They showed that β-actin is essential for the maintenance of mDNA transcription and mitochondrial membrane potential. The absence of β-actin impaired oxidative phosphorylation and ATP levels.

Moreover, the cytoskeleton interacts with mitochondria and is involved in the mitochondrial dynamics of fission and fusion. Mitochondrial fission occurs through membrane remodeling initiated by the recruitment of the GTPase dynamin-related protein, which requires the action of F-actin associated with the endoplasmic reticulum [11]. On the other hand, the fusion of outer mitochondrial membrane is controlled by proteins mitofusins 1 and 2, and the fusion of the inner mitochondrial membrane is controlled by protein optic atrophy 1 [12]. Mitochondria actually play an important role in cell survival and death. Damaged mitochondria undergo autophagy, a mitophagy that selectively targets mitochondria [13].

Autophagy is a process in which actin filaments participate in the formation of the carrier for autophagic vesicle formation by building the omegasome membrane, attaching phagophores to the endoplasmic reticulum and releasing the autophagosome, leaving behind an actin comet tail [14]. Thus, actin fills the space of endosomes, lysosomes, and autophagosomes in the endomembrane system of the cell.

One of the noninvasive modulators of cell proliferation and motility is light. The response of cells depends on the wavelengths, the dose of light, and the photosensitive molecules that accumulate in the cells. In the present study, photobiomodulation (PBM) represents the application of low amounts of light in the near-infrared range. This type of low-level laser therapy has been widely used for healing processes and has recently come to the forefront for use in cells with mitochondrial dysfunction due to interesting properties [15,16,17]. The mechanism of PBM is likely based on photon absorption by cytochrome c oxidase [18]. In other words, the stimulation of mitochondrial signaling pathways, for example, autophagy, by PBM is caused by the activation of complex IV of the mitochondrial electron transport chain (cytochrome c oxidase). Our recent studies have shown that PBM has effects on healthy and cancer cells [19,20]. Damaged mitochondria were remodeled by PBM in a very short time (within 4 h) after the irradiation of cells [20]. An indirect effect of PBM on the phosphorylation of protein kinase Cδ, which is involved in cell survival, was detected [21]. Recently, de Magalhaes et al. reported that PBM can alter the morphological characteristics of cells and their cytoskeleton [22]. Mokoena et al. showed that α-smooth muscle actin increased in an injured model after stimulation by PBM [23].

The delivery of light-sensitive molecules into cells enables the use of photodynamic therapy (PDT) to treat damaged cells. PDT is composed of three main factors: a photodynamically active drug (photosensitizer), visible light of an appropriate wavelength corresponding to the absorption maxima of the photosensitizer, and molecular oxygen in the target cells [24]. Photosensitizers have the ability to accumulate in cancer cells at significantly higher concentrations than in normal cells [25]. These properties have been exploited in the PDT-induced treatment of many types of tumors and their surrounding vasculature [26,27]. However, PDT also has several non-oncological applications, including for dermatological, respiratory, and ophthalmological diseases [28]. Nowadays, clinical trials of PDT are still ongoing.

It has been observed that PDT can effectively modulate various components of the cytoskeleton such as the actin and tubulin networks [29]. Malohlava et al. performed experiments focusing on morphological changes in HeLa cells after PDT treatment [30]. They observed the reorganization of the actin filament network and shortened microfilaments in PDT-treated cells compared to controls.

One of the photosensitizers studied is hypericin, which belongs to a group of anthraquinones that have a hydrophobic property [31]. Hypericin is also known to be a regulator of protein kinase C [32,33,34]. Because protein kinase C is involved in the phosphorylation of myosin, hypericin can also be expected to have effects on the actin network. In particular, photoactivated hypericin can have destructive effects on β-actin. Suloglu et al. reported that light-activated hypericin alters the organization of F-actin in colon adenocarcinoma cells [35].

Despite the high affinity of photosensitizers to cancerous tissue, some of these molecules can also be absorbed by surrounding non-cancerous tissue. In our previous study, we showed that combined treatment with PBM and PDT stimulated autophagy and apoptosis in human dermal fibroblasts. However, these effects were less pronounced in noncancerous cell lines than in cancer cells [19].

In recent years, researchers have increased their interest in the combination of PBM and PDT. PBM has been used as an adjuvant treatment to PDT of cancer in the oral cavity and this combination has reduced the extent of oral mucositis in patients [36,37,38]. The combination of antibacterial PDT and PBM has proven to be crucial in the treatment of cytomegalovirus associated with graft-versus-host disease [39].

Moreover, PBM has been shown to influence PDT by increasing the uptake of metal-based photosensitisers [40] and Photogem^®^ [41] by target cells and the homogenised production of protoporphyrin IX in vitro and in vivo [42]. Furthermore, the induction of autophagy in cancer cells by PBM led to an improvement of anti-cancer PDT in glioblastoma [19] and breast cancer cells [43].

In this study, we sought to improve the experimental protocol to reduce the side effects of PDT on healthy cells and investigate the effects of PDT and PBM on the cytoskeletal structure and mitochondrial metabolism of non-cancer cells.

## 2. Materials and Methods

### 2.1. Preparation of Cell Cultures

Dermal fibroblasts from HDFs (human dermal fibroblasts, the cell culture was obtained from Cell, Applications, San Diego, CA) were prepared in complete medium RPMI 1640 (Biosera, Nuaille, France). Glioblastoma from human U87 MG (Cells Lines Services, Eppelheim, Germany) were prepared in complete high glucose–pyruvate medium DMEM (Dulbecco’s modified Eagle medium with GlutaMAX™, Gibco-Invitrogen, Life Technologies Ltd., Paisley, UK). A supplement of 10% FBS (foetal bovine serum) and 1% penicillin/streptomycin (*w*/*w*) from Gibco-Invitrogen (Life Technologies Ltd., Paisley, UK) was added to the medium. Cell cultures were grown in flasks or Petri dishes (5% CO_2_ at 37 °C, in the dark dark, at 100% humidity) until 80–90% confluency was reached.

### 2.2. Therapeutical Protocols

**Figure 1:**
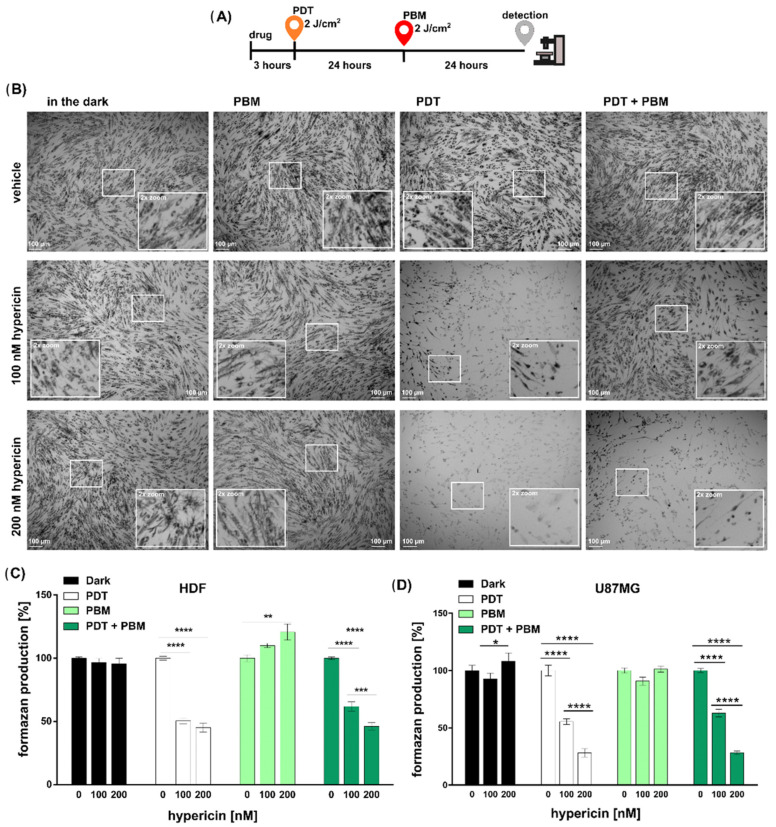
(**A**) Scheme of experimental protocols. (**B**) Bright-field images of HDF cells subjected to treatment (as denoted in figures) and incubated with MTT reagent (dark crystals). Results of MTT assay detected in (**C**) HDF and (**D**) U87MG cells. The data were relativized to the negative control. Data are presented as the mean ± SEM. Two-way ANOVA, Tukey’s posthoc test, * *p* < 0.05, ** *p* < 0.01, *** *p* < 0.001, **** *p* < 0.0001, comparison of irradiated groups between the control (0 nM hypericin) and hypericin-treated cells (100 nM hypericin).

To treat the cells, 100 or 200 nM hypericin (Sigma-Aldrich, Darmstadt, Germany) dissolved in DMSO or a vehicle of 100% dimethyl sulfoxide (DMSO, Sigma-Aldrich) was administered as the DMEM solution (DMSO < 1%) in the absence of light. After 3 h, the medium containing hypericin was removed, and a fresh complete medium was added to the cells. The cells were then treated with PDT (590 ± 10 nm light emitting diodes, 2 min, 2 J/cm^2^, 16.7 mW/cm^2^) and kept in the dark for 24 h. Following this, the cells were treated with PBM induced with a light dose of 2 J/cm^2^ (185 s with an irradiance of 10.8 mW/cm^2^). PBM was applied after 24 h of PDT treatment (see scheme in Figure 1A). The PBM protocol was achieved by the application of light at 808 nm (infrared diode laser, Changchun New Industries Optoelectronics Tech. Co. Ltd., Changchun, China) joined with a frontal diffuser of light (Medlight SA, Ecublens, Switzerland) to reach the illumination area (45 cm^2^, 675 mW). The evaluation of the processes occurring in the treated cells was performed 24 h after PBM treatment (see scheme in Figure 1A).

### 2.3. Cell Metabolism/Viability Assay

For the cell metabolism assay, 96-well plates were used. The therapeutical protocols were applied as described above. The principle of this method was the conversion of 3-(4,5-dimethylthiazol-2-yl)-2,5-diphenyltetrazolium bromide (MTT, Sigma-Aldrich, St. Louis, MO, USA) in purple formazan by active mitochondria. The MTT assay was performed according to the manufacturer’s instructions. After 3 h of incubation with the MTT reagent, formazan was dissolved in 100% DMSO and samples were colorimetrically detected at 560 nm (absorption) using a plate reader (GloMax^TM^-MultiDetection system with Instinct Software, Madison, WI, USA). These measurements were performed in triplicate.

Representative bright-field images of HDF cells with formazan crystals were detected with an Olympus microscope (Olympus IX70, Tokyo, Japan) using a 4× magnification dry objective (UPlanFLN 0.13 PhP, Olympus) and a digital camera.

### 2.4. Confocal Fluorescence Microscopy

Petri dishes for confocal fluorescence microscopy were used (SPL, Pocheon-si, Republic of Korea). When the cells reached 80% confluency, a therapeutical protocol was performed. Cell structures were labeled 24 h after PBM treatment or control dark conditions. Mitochondria were labeled after 15 min with 5 μM Rhodamine 123 (Rh123, Sigma-Aldrich, Darmstadt, Germany). Rh123 was excited at 488 nm and its emission was detected from 490 to 560 nm. Hypericin fluorescence was detected at >590 nm by excitation at 555 nm.

Immunofluorescence: treated HDF cells were fixed with 3.7% paraformaldehyde (Centralchem, Bratislava, Slovakia) in PBS for 7 min and rinsed 3× with a 4 °C cold phosphate saline buffer (PBS, Sigma Aldrich, Darmstadt, Germany). Subsequently cells were incubated with a −20 °C acetone/methanolic (Centralchem, Bratislava, Slovakia) solution (1:1 *w*/*w*) for 5 min at −20 °C and rinsed 3× with cold PBS. Cells were blocked with 5% bovine serum albumin (BSA, Sigma Aldrich, Germany) and a skimmed milk PBS solution for 1 h at room temperature. Cells were rinsed with cold PBS and primary antibodies (1:1000 anti-β-actin ab8227 and 1:1000 anti-ubiquinol-cytochrome C reductase ab110252, both purchased from abcam, Cambridge, UK) dissolved in 2.5% BSA and incubated overnight at 4 °C. Cells were rinsed 3× with cold PBS and incubated for 1 h at room temperature with secondary antibodies: anti-mouse AlexaFluor 546 (ThermoFisher Scientific, Waltham, MA, USA; conjugates to cytochrome b reductase; excitation 555 nm and emission detected in the range >560 nm) and anti-rabbit AlexaFluor 488 (abcam, UK; conjugates to β-actin; excitation 488 nm and emission detected in the range 500–550 nm) diluted in a 1% BSA solution. Cells were rinsed 3× with cold PBS. Cells were finally mounted with mounting medium Fluoroshield^TM^ (Sigma-Aldrich).

Confocal fluorescence images were collected with an LSM700 confocal microscope (ZEISS, Oberkochen, Germany) as described previously [19]. The analysis was conducted with ZEN2011 software, version 1.0 (ZEISS).

The intercellular free area measurement was conducted using ImageJ software (Image J, version 1.54d). We utilized the “Freehand selection” tool to delineate the boundaries of the intercellular free area by drawing or outlining shapes.

Following image analysis, the total area of the intercellular spaces was calculated by summing up all the shapes representing these areas. This summation process was executed using Excel (Microsoft 365 Apps for enterprise, version 2311).

### 2.5. Stimulated Emission Depletion (STED) Microscopy

HDF cells were grown and treated for confocal microscopy. The secondary antibodies were replaced in STED by STAR ORANGE (aberior) for β-actin and STAR RED (aberior) for cytochrome b reductase suitable for STED microscopy. Superresolution images were acquired with a stimulated emission depletion (STED) imaging system (STEDYCON, abberior Instruments GmbH, Göttingen, Germany) with a 100× objective (ZEISS alpha Plan Apo 100× 1,46 Oi). An excitation laser of 640 nm was used for STAR RED dye and a 561 nm laser was used for STAR ORANGE dye. Depletion was performed with a 775 nm STED laser. Nuclei labeled with Hoechst were excited at 405 nm and imaged in confocal mode only. Emission was detected at 660 nm, 616 nm, and 461 nm, respectively. Collected images were processed in ImageJ software [44].

### 2.6. Statistical Analysis

In our study, a two-way ANOVA was employed to concurrently assess the influence of two categorical independent factors—hypericin and light condition—on the intercellular free area. This analysis not only evaluated the main effects of each factor but also scrutinized their interaction effect (see Supplementary Materials). Understanding this interaction was crucial as it elucidated whether the combined impact of hypericin with PDT or PBM simultaneously affected the intercellular free area differently than their individual effects alone.

The cell viability of HDF and U87MG cells, mitochondrial distribution, and intercellular free area in HDF cells were analyzed by two-way ANOVA (the factors used in analysis were hypericin treatment and light condition). Differences between treatments were compared by post hoc Tukey multiple comparisons tests. Analysis of the intercellular area of HDF cells was supplemented with Student’s *t*-test. Significant differences were taken into consideration at *p* < 0.05. The average ± standard error of the mean (SEM) was finally presented.

### 2.7. An Overview of the Study Method with an Emphasis on Relevant Publication Trends

In this work, we also added a basic methodological data mining component that focused on summarizing publication datasets to strengthen our hypotheses, themes, and subthemes. By observing changes in publication trends of articles retrieved from the PubMed database, we aimed to investigate the themes of specialized studies, among others, in our study. We began the data mining process with 36 logical PubMed queries that combined title and abstract data with specific time spans. These queries defined three distinct, non-overlapping time periods: 2008–2013, 2013–2018, and 2018–2023, each starting on 1 January and ending on 1 January of the following year. We searched for two different types of therapies in this dataset: “photodynamic therapy” and “photobiomodulation therapy”. At the same time, our searches focused on cellular components, particularly those connected to mitochondrial metabolism and function. Our search also included the cytoskeleton, healthy or non-cancerous cells, and cancerous cells or tumors, with a focus on fibroblasts and skin. We were aware of possible errors in our selection process, such as not including all synonyms or excluding papers that were already included in other searches. This simplification was based on the belief that trends retain their significance despite the obvious inaccuracies in population numbers. After assessing successive relative increases and identifying the most important ones, we compiled a set of occupationally relevant cases. For these cases, we created time dependencies that allowed for a more detailed annual evaluation. Given the substantial differences in the number of publications between PDT and PBM, we were able to identify the most important trends using a logarithmic scale on the axis of publications.

## 3. Results

### 3.1. The Metabolic and Structural Alteration of Mitochondria Induced by Light

HDF cells were subjected to four different regimens: dark conditions or PBM, PDT, or PDT + PBM treatment, the temporal sequence of which is schematically shown in Figure 1A. These regimens were chosen to modulate the metabolic activity of mitochondria stressed with the known protein kinase C inhibitor hypericin in the dark and the activator after light application [21,33,34]. Hypericin has been used in PDT as a photosensitizer with promising properties for diagnosing and curing cancer with PDT [45,46,47,48,49,50,51]. The main advantages of PDT are its high cancer specificity and minimal toxicity compared with healthy cells. The HDF cells in the present study represented healthy cells that could absorb hypericin, but the application of light here was expected to be less effective than in cancer cells.

The confluence and organization of HDF cells before and after treatment were highlighted with the MTT reagent (dark crystals), shown in Figure 1B. A marked inhibition of formazan production and large gaps between cell assemblies were observed after hypericin and PDT treatment. In cells irradiated with PBM (without pretreatment with PDT), large formazan crystals were found. This effect reflects the increase in mitochondrial dehydrogenase activity and consequently higher metabolic activity of the cells. Interestingly, the photodamage effect induced by PDT was significantly reduced when PBM was applied to cells pretreated with hypericin and PDT (Figure 1B).

Moreover, the viability and activity of mitochondria reflected the metabolic activity of cells. To analyze the rate of metabolic activity of fibroblasts (HDF in Figure 1C) and glioblastoma cells (U87MG in Figure 1D), an MTT assay was performed. Two-way ANOVA tests showed a significant interaction between hypericin administration and irradiations in HDF cells (F _(6, 139)_ = 24.43; *p* < 0.0001) and U87MG cells (F _(6, 143)_ = 30.95; *p* < 0.0001). As expected, hypericin-induced photodynamic therapy significantly reduced metabolic activity in both cell lines compared to controls (Tukey’s post hoc test, *p* < 0.0001). A significant reduction of metabolic activity was seen after hypericin-treated cells (HDF and U87MG) were exposed to combined irradiation (PDT + PBM) in a dose-dependent manner (Tukey’s post hoc test, *p* < 0.001 and *p* < 0.0001, respectively). In addition, cell metabolism was increased in hypericin-treated HDF cells (Figure 1C) compared to the corresponding control (0 nM hypericin) in the PBM-irradiated group (Tukey’s post hoc test, *p* < 0.01), but not in glioma cells (Figure 1D). Our analysis also compared the effects of hypericin-induced PDT and combined therapy (PDT and PBM) on cell metabolism. Hypericin-induced photodynamic therapy significantly reduced metabolic activity not only compared to the control but also compared to combined therapy (PDT and PBM) in HDF cells (Tukey’s post hoc test, *p* < 0.01). On the other hand, the lower level of metabolic activity caused by hypericin-induced PDT compared to the control was not significantly increased by subsequent PBM irradiation in U87MG cells.

**Figure 2:**
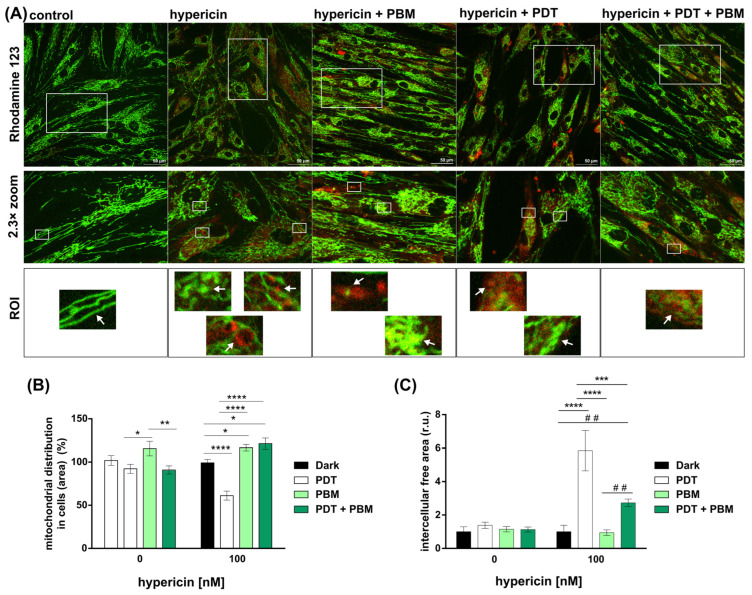
(**A**) Fluorescence images of Rhodamine 123 (green) and hypericin (red) in live HDF cells subjected to treatments (as denoted in figures). Zoom images and regions of interest (ROI) were selected. White arrows point to mitochondria structure. (**B**) Analysis of cellular area filled with mitochondria. (**C**) Analysis of intercellular area (r.u.—relative unit). The data were relativized to the negative control. Data are presented as the mean ± SEM. Two-way ANOVA, Tukey’s posthoc test, * *p* < 0.05, ** *p* < 0.01, *** *p* < 0.001, **** *p* < 0.0001, comparison of irradiated groups between the control (0 nM hypericin) and hypericin-treated cells (100 nM hypericin). Student *t* test, ## < 0.01.

The mitochondrion is a bi-membrane organelle that is highly dynamic and forms an extensive branching network in most mammalian cells. Rhodamine 123 can be localized in the intermembrane space of mitochondria and often reflects the state of mitochondrial membrane potential. Representative confocal fluorescence images of HDF cells subjected to treatment and labeled with Rhodamine 123 are shown in Figure 2A. Hypericin as a fluorescent molecule had a red fluorescence that can be identified in the corresponding images (Figure 2A). Deeper analysis of selected regions of interest (ROI) revealed distinct differences in mitochondrial structure. Whereas mitochondria from HDF cells treated with the vehicle were tubular, fused, and straight, the administration of hypericin resulted in the curvature of these structures (zooms in Figure 2A). Hypericin-loaded vesicles were found to be entangled with mitochondria (red-stained vesicles in the zooms of Figure 2A). The application of PBM to cells increased the number of mitochondria and forced their fusion. In contrast, PDT triggered fission and a reduction in mitochondria. The combined treatment of PDT and PBM reversed the latter effect of PDT. Although, PBM increased the occurrence of hypericin-loaded vesicles in HDF cells.

Our results showed that the distribution of mitochondria could be altered by the interaction between the application of hypericin and the irradiation conditions (PDT: at 590 nm, PBM: at 808 nm) in HDF cells (two-way ANOVA, F _(3, 85)_ = 9.841, *p* < 0.0001). The post hoc analysis showed that cells treated with 100 nM and then irradiated at 590 nm had significantly lower mitochondrial distribution compared to the control (dark conditions) and other irradiated groups (PBM, PDT + PBM) (Figure 2B) (Tukey’s post hoc test, *p* < 0.0001). After hypericin-induced PDT, mitochondria were mainly localized around the nuclei (Figure 2A). On the other hand, the mitochondrial area was significantly larger after PBM and combined therapy (PDT + PBM) in the presence of hypericin compared to the control (Figure 2A,B) (Tukey post hoc test, *p* < 0.05). Although the analysis also showed significantly greater expansion of mitochondria in the PBM group without the presence of hypericin compared to PDT (*p* < 0.05) and combined PDT + PBM treatment (*p* < 0.01), there was no significant difference compared to the non-irradiated control group.

The connection of cells in a population is very important for inter- and intracellular communication. The free space between cells was analyzed, and significantly large gaps were found in the hypericin-induced PDT and PDT + PBM groups (Figure 2C). However, the application of PBM after PDT resulted in a significant decrease in empty space (Figure 2C). This may indicate increased cell motility and cytoskeletal reorganization.

### 3.2. The Organization of β-actin in HDF Cells Subjected to PDT and PBM

The structure of the cytoskeleton of HDF cells consists of tubulin and actin filaments. These filaments are tangled and organized according to the needs of the cell. Different filaments respond to cell movement and others respond to stress. A long-term study of hypericin-induced PDT in our laboratories found that β-actin, similar to glyceraldehyde 3-phosphate dehydrogenase, responded significantly to PDT [20]. This was an attempt to follow the organization of β-actin in adult HDFs, which may undergo senescence along with other processes.

**Figure 3:**
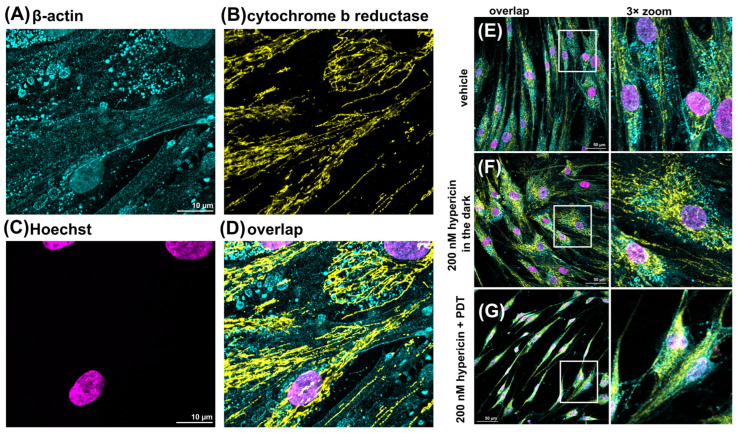
Immunostaining of HDF cells against (**A**) β-actin (AlexaFluor 488), (**B**) cytochrome b reductase (AlexaFluor 546), (**C**) Hoechst, and (**D**) overlap. HDF cells were treated with (**A**–**E**) vehicle (DMSO), (**F**) hypericin in the dark, and (**G**) hypericin-induced PDT. Images were zoomed to better recognize mitochondria and actin fibers.

Figure 3A shows the typical organization of β-actin (cyan) in adult HDFs. Note that the positive fluorescence in the nuclei was a false fluorescence from Hoechst (Figure 3C). The mitochondria in Figure 3B were identified by cytochrome b reductase (yellow). A very specific localization of the two proteins studied was observed upon overlap (Figure 3D). For this reason, the overlapping images were further visualized. Figure 3E–G show β-actin in spherical structures and various fibers extending along the size of the cells and intertwined with mitochondria. HDF cells treated with PDT with 200 nM hypericin exhibited a sharp, elongated shape with β-actin, shown in Figure 3G. Large gaps were observed in the population of these cells, but the remaining cells were interconnected by β-actin fibers.

**Figure 4:**
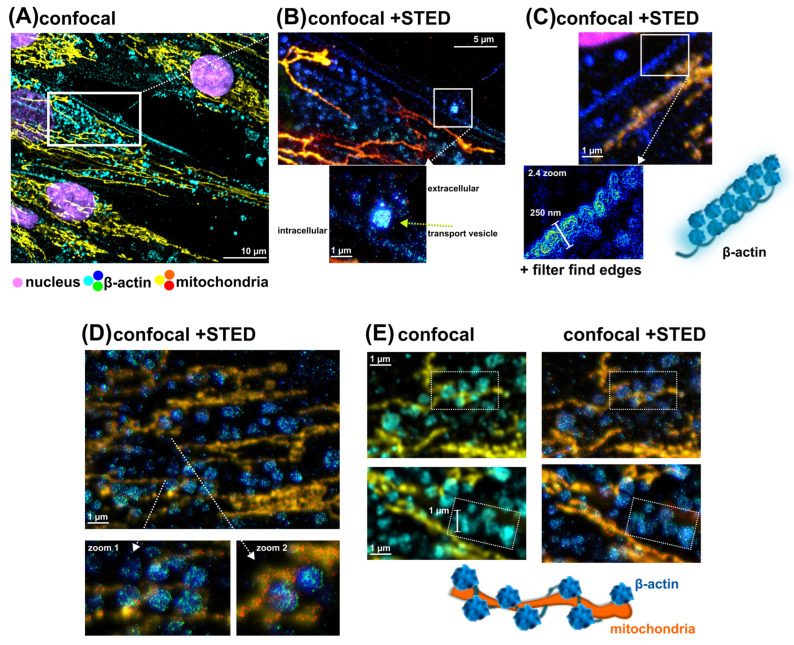
Confocal fluorescence and STED images of HDF cells (treated with vehicle). (**A**) Immunofluorescence against the β-actin (STAR ORANGE, green, cyan, blue), cytochrome b reductase (STAR RED, yellow, orange, red), and Hoechst (magenta). Special selections were focused on (**B**) cellular transport, (**C**) plasma membrane area, (**D**,**E**) mitochondria, and β-actin interaction. Organization of β-actin granules and bundles was proposed according to images (white dashed rectangular boxes in the figure).

Thanks to the super-resolution technique of STED imaging, the structure of β-actin bundles can be detected. Figure 4 shows the differences between confocal fluorescence images and enhanced STED images. In STED, the localization of detected proteins was detected by immunolabeling with specific secondary antibodies for STED (STAR ORANGE and STAR RED), which were recognized as red spots (cytochrome b reductase) in mitochondria (yellow) and green spots (β-actin) in actin bundles (blue). It should be noted that the yellow and red colors corresponded to the same proteins, but the resolution was better detected with STED due to the Rayleigh criterion of resolution.

Thanks to the STED images, the intercellular transport (Figure 4B) between HDF cells could be observed. The detected vesicles active in cellular transport consisted of β-actin. It should be noted that hypericin-loaded vesicles were frequently detected in live HDF cells after hypericin administration (Figure 1B).

Two main types of β-actin bundles were observed (also in vehicle-treated HDF cells) with long, thin fibers and granular structures of different sizes. The first type of β-actin bundle is shown in Figure 4C. This bundle looked like a thin (280 nm) spiral, and closer inspection with STED and post-processing of the image (application of an edge-finding filter) revealed the repeated appearance of small spherical β-actin structures, as shown in the schematic diagram. Several subunits could be identified. However, other techniques should be used in the future for better identification.

Larger spherical structures of β-actin were recognized in Figure 4D,E. These structures also formed large, robust (1 µm) spirals that supported mitochondria. The organization of these structures is suggested in Sheme E (Figure 4E). The close association of mitochondria and β-actin granules can be seen in Zoom 2 of Figure 4D.

Further, these imaging techniques were applied to visualize the change in mitochondrial and β-actin structures in adult HDF cells exposed to 100 nM hypericin in the dark, PBM, and PDT. All of these treatments did not result in the disruption of subcellular structures and cell morphology. However, the organization of β-actin was altered and it was recognized to be specifically involved in mitochondrial reorganization.

**Figure 5:**
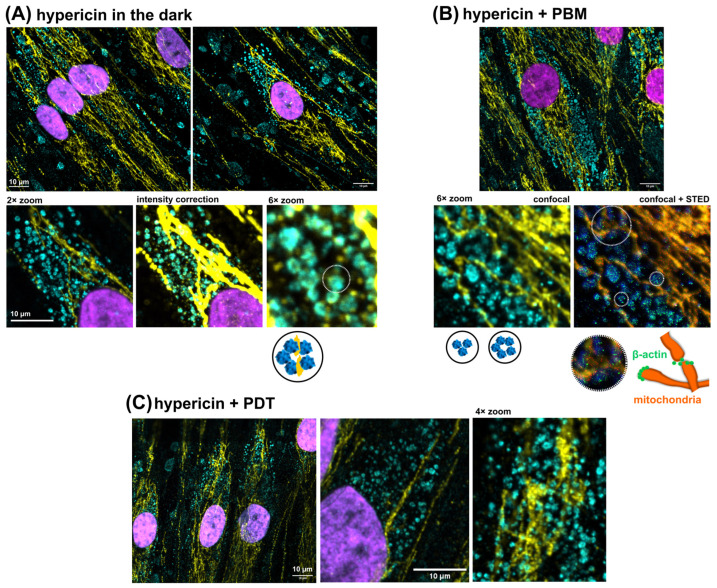
Immunofluorescence against the β-actin (STAR ORANGE, green, cyan, blue), cytochrome b reductase (STAR RED, yellow, orange, red), and Hoechst (magenta) in HDF cells subjected to (**A**) 100 nM hypericin in the dark, (**B**) 100 nM hypericin + PBM, and (**C**) 100 nM hypericin + PDT. Organization of β-actin granules and mitochondria was proposed according to images (area of interest was selected with dotted circle).

Representative HDF cells treated with 100 nM hypericin and kept in the dark are shown in Figure 5A. Long mitochondria and their dense abundance in the perinuclear region were observed. Many globular β-actin structures were also found in this region. Upon deep inspection, short mitochondria were found in close proximity to the β-actin arrangement of granules. Regularly, five units of β-actin granules were found forming a large sphere connected to the mitochondria (see Figure 5A). Several of these pentads followed a row in which short mitochondria were found. This looked like the origin of a future long-fused mitochondrion.

Compared with the cells kept in the dark, the cells treated with hypericin and PBM consisted of a large number of smaller spherical β-actin units forming groups of three or five (see Figure 5B). However, in some locations, β-actin spots detected by STED were stored at the end/beginning of long mitochondria (see image and illustration in Figure 5B). In general, the distribution of spherical β-actin structures was more widespread than in the cells under dark conditions. Mitochondria in PBM-treated cells formed straight, long networks.

Photodamage induced by hypericin and PDT resulted in the twisting of mitochondria (Figure 5C). The β-actin structures looked more like a disintegrated and disoriented distribution. Because hypericin can be localized in such structures/vesicles, oxidative stress induced by hypericin–PDT could lead to the partial destabilization of β-actin structures.

## 4. Discussion

The HDF cells in this study represented a model of healthy cells exposed to light under very specific conditions. Hypericin-induced PDT, a targeted cancer treatment, was applied to HDF cells to investigate the extent of eradication. HDF cells were grown in a confluence characterized by close association of the cells. PDT caused photodamage to the tight junction and opened the space between the cells (Figure 1B and Figure 2C).

We previously observed HDF cells exposed to PBM and hypericin-induced autophagy [19]. However, the subsequent application of PDT converted this rescue process to apoptosis. In the present work, the administration times were reversed with the aim of rescuing damaged subcellular organelles. PBM was administered 24 h after PDT. This combination resulted in a reduction in intercellular space (Figure 1B and Figure 2C). In addition, PBM increased the metabolic activity of HDF cells exposed to hypericin and, most importantly, inhibited the effect of PDT on the cells (Figure 1C) and stimulated mitogenesis (Figure 2B).

Microscopic analysis of the combined treatment revealed that the shape of the cells and mitochondrial network was altered by PBM (Figure 2A). Whereas mitochondrial fission was mostly observed after hypericin-induced PDT, both hypericin in the dark and PBM alone or in combination with PDT triggered mitochondrial fusion. Because mitochondria are the main source of ATP, mitochondrial fusion suggests that cells have a huge energy demand.

Li et al. reported that PBM ameliorated the imbalance of mitochondrial fusion in spinal cord tissue in the subacute phase, reduced neuronal cell death, and improved the motor function of rat hind limbs in a time-dependent manner [52]. They observed that PBM reduced the levels of mitochondrial fission 1 protein, mitochondrial fission factor, and dynamin-related protein 1.

Using human adipose mesenchymal stem cells, Pan et al. showed that PBM can modulate mitochondrial membrane potential, oxidative stress level, and vesicle transport as a promising treatment for cell migration, proliferation, and differentiation [53].

The actin cytoskeleton is strongly involved in mitochondrial function and dynamics [8]. In hypericin-treated cells, many structures that can be identified as G-actin oligomers were found in the dark (Figure 5A) and after PBM (Figure 5B), where mitochondrial fusion was detected. Moreover, the close contact of G-actin with short mitochondria was observed in these cells. They were in a position resembling a string of beads. Moreover, the strong support of mitochondria by G- and F-actin fibers straightened mitochondrial branches in a large network.

The inhibition of PKC could result in the inhibition of myosin phosphorylation and activity [6]. This, in turn, could affect the contractility of the cell. In contrast to PBM, PDT caused the disaggregation of G- and F-actin, accompanied by twisting and curvature of the mitochondrion (Figure 5C). Moreover, higher doses of hypericin and PDT could lead to the complete destabilization of the cell and the fission of mitochondria [20,33].

Suzuki et al. investigated the effect of talaporfin sodium-based PDT on human umbilical vein endothelial cells [54]. They focused on the dynamics of tubulin and F-actin. PDT applied to the cells resulted in the destruction of endothelial tubules, depolymerization of microtubules, induction of F-actin stress fiber formation, and phosphorylation of the myosin light chain.

The results presented in this work are intended to support the view that β-actin is a sensitive factor controlling mitochondrial dynamics that changes during light application (PBM and PDT).

Indeed, PBM, PDT, and their combination have garnered great attention from scientific community. Table 1 summarizes the works in which various photosensitizers were investigated regarding PBM and PDT activities applied to different types of diseases.

**Figure 6:**
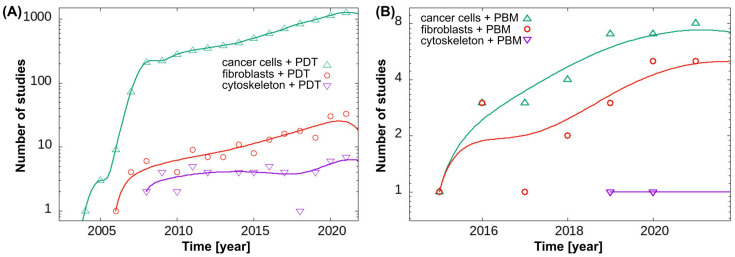
Trends in publications related to (**A**) PDT and (**B**) PBM applied to cancer cells and fibroblasts and in which the cytoskeleton was studied.

Interest in the application of PBM and PDT to fibroblasts and cancer cells has increased over the past few decades (see Figure 6). We have also focused on work that has examined the cytoskeleton after application of PDT and PBM. There is a large difference between the number of published papers related to PDT in cancer cells compared to fibroblasts (Figure 6A), but a small difference in the number of papers that applied PBM (Figure 6B). Among these studies, we identified papers in which the cytoskeleton was affected by PDT, and only a few were related to PBM. Perhaps the cytoskeleton is not the first target for treatment, but our work shows that it is important to monitor it also because of its involvement in cell–cell communication and interaction.

## 5. Conclusions

In conclusion, this study explored changes in intercellular communication, mitochondrial structures, and cytoskeletal dynamics, particularly focusing on the role of β-actin. The findings revealed that exposure to PDT led to the disintegration of β-actin oligomers, while PBM increased their formation. Additionally, interactions between β-actin and mitochondria were observed. The combination of PDT and PBM treatments was highlighted as significant in reducing the potential side effects of cancer treatment using PDT on healthy cells, as indicated by cell metabolism assays. Overall, this work underscores the importance of β-actin as a key parameter in understanding how cells respond to PDT and PBM, shedding light on potential strategies to mitigate the impact of light-induced stress on cellular structures and functions.

## Figures and Tables

**Table 1:** Summary of works in which PBM and PDT were combined.

Photosensitizer	PBM Wavelength	PDT Wavelength	Model	Disease	Reference
			In Vitro/In Vivo		
curcumin	660 nm	* 468 nm	human oral cavity	oral mucositis	[36]
			human intravascular blood		
curcumin	660 nm	* 468 nm	human oral cavity	oral mucositis	[55]
hypericin	* 808 nm	590 nm	U87 MG	glioblastoma multiforme -	[19]
			HDF		
zinc tetrasulfonic acid phthalocyanine (ZnPcS4)	681 nm	680 nm	MCF-7	breast cancer	[43]
methylene blue	660 nm (0.3 J, 3 s per point)	* 660 nm (5 J, 50 s per point)	human oral cavity	oral cytomegalovirus infection	[39]
ruthenium complexes [Ru(Pc)] or trans-[Ru(NO)(NO2)(Pc)]	850 nm	* 660 nm	A375	melanoma	[40]
Photogem^®^	* 780 nm	630 nm	SCC-25 and SCC-4	head and neck squamous cell carcinomas	[41]
curcumin	660 nm	* 468 nm	human oral cavity	oral mucositis	[37]
methylene blue	660 nm (1 J, 10 s per point	* 660 nm (2 J, 20 s per point)	human oral cavity	osteoradionecrosis	[38]
	808 nm (2 J, 20 s per point)		top of the major salivary glands	xerostomia	

* indicates the treatment that was applied first.

## Data Availability

Data are presented in the text.

## Supplementary Materials

The following supporting information can be downloaded at: https://www.mdpi.com/article/10.3390/pharmaceutics16010020/s1, Figure S1: Table displaying the representative results of the two-way ANOVA.
