# Supplementary material for: The Remodulation of Actin Bundles during the Stimulation of Mitochondria in Adult Human Fibroblasts in Response to Light
Source: Pharmaceutics. 2023 Dec 22;16(1):20. doi: 10.3390/pharmaceutics16010020 (PMC10818370; doi:10.3390/pharmaceutics16010020)
Supplement: Supplementary file 1 [file pharmaceutics-16-00020-s001.zip › pharmaceutics-2721853-supplementary.pdf]

Supplementary Materials

Remodulation of actin bundles during stimulation of mitochondria in adult human fibroblasts in response to light

Soňa Olejárová [1], Denis Horváth [2] and Veronika Huntošová [2,*]

[1] Department of Biophysics, Institute of Physics, Faculty of Science, P.J. Šafárik University in Košice, Jesenná 5, 041 54 Košice, Slovakia; sona.olejarova@student.upjs.sk
[2] Center for Interdisciplinary Biosciences, Technology and Innovation Park, P.J. Šafárik University in Košice, Jesenná 5, 041 54 Košice, Slovakia; denis.horvath@upjs.sk; veronika.huntosova@upjs.sk;
* Correspondence: veronika.huntosova@upjs.sk;

In our study, a two-way ANOVA was employed to concurrently assess the influence of two categorical independent factors—hypericin and light condition—on the intercellular free area. This analysis not only evaluates the main effects of each factor but also scrutinizes their interaction effect. Understanding this interaction is crucial as it elucidates whether the combined impact of hypericin with PDT or PBM simultaneously affects the intercellular free area differently than their individual effects alone.

**Two-way ANOVA**	Ordinary				
Alpha	0.05				
**Source of Variation**	**% of total variation**	**P value**	**P value summary**	**Significant?**	
Interaction	28.78	0.0004	***	Yes	
Row Factor	18.24	0.0004	***	Yes	
Column Factor	36.97	<0.0001	****	Yes	
**ANOVA table**	**SS (Type III)**	**DF**	**MS**	**F (DFn, DFd)**	**P value**
Interaction	20.93	3	6.975	F (3, 17) = 10.41	P=0.0004
Row Factor	13.26	1	13.26	F (1, 17) = 19.78	P=0.0004
Column Factor	26.88	3	8.961	F (3, 17) = 13.37	P<0.0001
Residual	11.40	17	0.6703		

Figure S1: Table displaying the representative results of the two-way ANOVA.

We included a table displaying the results of the two-way ANOVA, providing a detailed overview. The F-value obtained from ANOVA compares the variation between group means to the variation within the groups. For instance, an $F(6, 143) = 30.95$ result indicates that the first number (6) signifies the degrees of freedom for the numerator, representing the variability between group means, while the second number (143) refers to the degrees of freedom for the denominator, indicating the variability within the groups.

The calculated F-value of 30.95 indicates the ratio of the variance between the group means to the variance within the groups. This statistical measure helps ascertain if the observed differences among means are statistically significant or might be due to random chance.
